# Supervisory Leaders in Aging: One-Year Practice Change Outcomes of Innovative Training Program

**DOI:** 10.1093/geroni/igaa057.062

**Published:** 2020-12-16

**Authors:** Daniel Kaplan, Barbara Silverstone, Keith Chan, Amanda Spishak-Thomas

**Affiliations:** 1 Adelphi University, Cortlandt Manor, New York, United States; 2 SBW Partners, Inc., New York, New York, United States; 3 State University of New York at Albany, Albany, New York, United States; 4 Columbia University, New York, New York, United States

## Abstract

Social services for older adults are instrumental in addressing vulnerabilities associated with aging. Yet, practitioners report needing expanded geriatric knowledge and enhanced supervision. Agency-based supervision is essential to skilled practice and staff retention, directly impacting the quality of services delivered by the teams they support. The Supervisory Leaders in Aging (SLA) program of the National Association of Social Workers (NASW) was designed to strengthen supervision of the social service workforce. The SLA program, adopted in four states (IL, FL, MD, and NY), trained 134 MSW supervisors who support 1,200 social service staff, aimed at enhancing the well-being of 264,000 clients annually. This paper reports newly available final outcomes data from the 3-year implementation study of SLA. Trainees self-rated use of relevant supervisory best-practices was measured with novel 30-item instrument which captured frequency in use of supervisory best practices. The measure was administered prior to the first session and at three and twelve months after the final session. Comparisons of ratings across time periods demonstrate a range of positive and significant increases at the end of program workshops (0.12–0.56; mean of 0.30 points) and after one year (0.18–0.53; mean of 0.34 points). Supervisory best practices were maintained by those who already engaged in these behaviors, and participants who previously underutilized best practices adopted and maintained these behaviors as a result of the workshops. Implications of this tested model for enhancing workforce capacity will be discussed, including variation of impacts by supervisor characteristics and retention of learning gains over time.

